# Combination of Linkage Mapping, GWAS, and GP to Dissect the Genetic Basis of Common Rust Resistance in Tropical Maize Germplasm

**DOI:** 10.3390/ijms21186518

**Published:** 2020-09-06

**Authors:** Maguta Kibe, Christine Nyaga, Sudha K. Nair, Yoseph Beyene, Biswanath Das, Suresh L. M, Jumbo M. Bright, Dan Makumbi, Johnson Kinyua, Michael S. Olsen, Boddupalli M. Prasanna, Manje Gowda

**Affiliations:** 1 International Maize and Wheat Improvement Center (CIMMYT), P. O. Box 1041-00621, Nairobi 00100, Kenya; maguta.kibe@gmail.com (M.K.); christinenyaga96@gmail.com (C.N.); Y.Beyene@cgiar.org (Y.B.); b.das@cgiar.org (B.D.); l.m.suresh@cgiar.org (S.L.M.); b.jumbo@cgiar.org (J.M.B.); D.Makumbi@cgiar.org (D.M.); M.Olsen@cgiar.org (M.S.O.); b.m.prasanna@cgiar.org (B.M.P.); 2 Jomo Kenyatta University of Agriculture and Technology (JKUAT), Nairobi 00100, Kenya; johnsonkinyua@jkuat.ac.ke; 3 International Maize and Wheat Improvement Center (CIMMYT), ICRISAT Campus, Patancheru, Greater Hyderabad 502324, India; Sudha.Nair@cgiar.org

**Keywords:** genome-wide association study, genomic prediction, joint linkage association mapping, genotyping by sequencing, resistance, common rust

## Abstract

Common rust (CR) caused by *Puccina sorghi* is one of the destructive fungal foliar diseases of maize and has been reported to cause moderate to high yield losses. Providing CR resistant germplasm has the potential to increase yields. To dissect the genetic architecture of CR resistance in maize, association mapping, in conjunction with linkage mapping, joint linkage association mapping (JLAM), and genomic prediction (GP) was conducted on an association-mapping panel and five F_3_ biparental populations using genotyping-by-sequencing (GBS) single-nucleotide polymorphisms (SNPs). Analysis of variance for the biparental populations and the association panel showed significant genotypic and genotype x environment (GXE) interaction variances except for GXE of Pop4. Heritability (*h^2^*) estimates were moderate with 0.37–0.45 for the individual F_3_ populations, 0.45 across five populations and 0.65 for the association panel. Genome-wide association study (GWAS) analyses revealed 14 significant marker-trait associations which individually explained 6–10% of the total phenotypic variances. Individual population-based linkage analysis revealed 26 QTLs associated with CR resistance and together explained 14–40% of the total phenotypic variances. Linkage mapping revealed seven QTLs in pop1, nine QTL in pop2, four QTL in pop3, five QTL in pop4, and one QTL in pop5, distributed on all chromosomes except chromosome 10. JLAM for the 921 F3 families from five populations detected 18 QTLs distributed in all chromosomes except on chromosome 8. These QTLs individually explained 0.3 to 3.1% and together explained 45% of the total phenotypic variance. Among the 18 QTL detected through JLAM, six QTLs, *qCR1-78, qCR1-227, qCR3-172, qCR3-186, qCR4-171*, and *qCR7-137* were also detected in linkage mapping. GP within population revealed low to moderate correlations with a range from 0.19 to 0.51. Prediction correlation was high with r = 0.78 for combined analysis of the five F_3_ populations. Prediction of biparental populations by using association panel as training set reveals positive correlations ranging from 0.05 to 0.22, which encourages to develop an independent but related population as a training set which can be used to predict diverse but related populations. The findings of this study provide valuable information on understanding the genetic basis of CR resistance and the obtained information can be used for developing functional molecular markers for marker-assisted selection and for implementing GP to improve CR resistance in tropical maize.

## 1. Introduction

Common rust (hereafter CR) caused by *Puccina sorghi* is one of the most destructive fungal foliar diseases in maize-growing regions predominantly in humid areas and has been reported to cause 12 to 61% yield losses in a favorable environment [1,2]. These yield losses are subject to leaf area infected and host growth stages whereby the former has been estimated to cause about 3–8% yield loss for each 10% of the total leaf area affected [3]. Quantitative resistance is due to partial resistance or adult plant resistance [4]. Numerous studies have suggested that older and mature tissues have more resistance to CR than younger soft tissues [4,5,6].

Past efforts to control CR through conventional means have been largely unsuccessful and also affected by unpredictable weather, and the use of fungicides leads to environmental effects and increased production costs [7]. Host-plant resistance has been identified as the most reliable and economically viable option among several available options to alleviate plant diseases [7,8]. In the case of CR, researchers have identified both qualitative and quantitative resistance [9,10]. Resistance through R genes has been identified more than 25 dominant race-specific (*Rp*) genes in chromosomes (chr) 3, 4, and 10 [9,11], which mediate the recognition of the pathogen and trigger a hypersensitive reaction to prevent further spread of the pathogen [12]. However, novel *P. sorghi* races can overcome the qualitative resistance in some genotypes and this requires a continuous search for sources of stable and durable resistance (quantitative) in order to manage the disease. Identifying resistant germplasm to CR and incorporation of resistant genes or genomic regions to elite lines and commercial hybrids has the potential to increase yields with lower production costs [13].

Genetic mapping through linkage analyses and GWAS have been used in many studies in plant breeding [12,14,15,16,17,18,19,20,21,22,23,24,25,26]. The two approaches exploit the recombination’s ability to break up the genome into fragments that can be correlated with phenotypic variation but differ with the type of control they have on the recombination [27]. Linkage mapping in plants utilizes biparental crosses and, thus, is a closed controlled system. This further limits the number of recombinations that can sufficiently shuffle the genome into small fragments and results into QTLs localized in large chromosomal regions [28]. On the other hand, GWAS uses natural populations that mimic historical recombinations and provide a higher resolution as compared to linkage mapping [25]. This approach, however, has no control over relatedness and is prone to spurious associations. Accounting for population structure and kinship relatedness in a mixed model has been the most effective method of reducing false associations in GWAS [29]. Quantitative resistance for CR, a more durable approach, has also been exploited in a few studies through quantitative trait loci (QTL) analysis and genome-wide association studies (GWAS). A total of 41 QTLs have been identified conferring resistance to CR across all maize chromosomes in four separate studies utilizing different maize germplasm [7,10,13,30]. One study by Olukolu et al. [12], carried out GWAS and identified three marker trait associations (MTAs) in chr 2, 3, and 8 using 274 diverse inbred lines and 246,497 SNPs. Another study combined linkage and association mapping on 296 tropical maize inbreds and identified 25 QTLs on chr 1, 3, 5, 6, 8, and 10 associated with CR resistance [31]. These markers and candidate genes were directly or indirectly involved with plant disease responses [12,31].

In cases where the phenotype is strongly correlated with relatedness, population mapping even with the use of mixed models can be severely underpowered [27]. Joint linkage association mapping (JLAM) has the potential to overcome the downsides of the both linkage mapping and association mapping approaches whereby one compliments the other. Association mapping will increase the mapping resolution power while linkage mapping will account for relatedness in cases where Q + K explains most of the phenotypic variance. To our knowledge, no study has been reported utilizing JLAM to identify QTLs conferring resistance to CR in maize.

Another promising genomic tool is the genomic prediction (GP) which has been applied successfully in many plant breeding studies [32,33]. Previous reports have indicated the potential of GP to increase genetic gain and reduce the time taken in breeding programs significantly [33]. GP utilizes genome-wide markers to estimate the effects of all loci and computes a genomic estimated breeding value (GEBVs) [34]. Unlike genetic mapping, GP does not identify significant MTAs first but goes ahead to use all markers available to estimate their effects thus providing a powerful approach to account for any effects that might have been missed by genetic mapping. However, this doesn’t mean complete withdrawal of genetic mapping but rather the incorporation of the two in genetic studies as complimentary approaches since each provide significant advantages. The main objectives of this study were to: (1) assess the phenotypic variation for CR by evaluating five segregating populations and an association panel in multiple environments; (2) detect stable and the novel genetic loci significantly associated with CR resistance by using QTL mapping, JLAM, and GWAS; and (3) GP within populations and Improved maize for African Soil (IMAS) panel and among the IMAS and F3 populations in order to understand the genetic architecture of maize CR resistance.

## 2. Results

**Figure 1:**
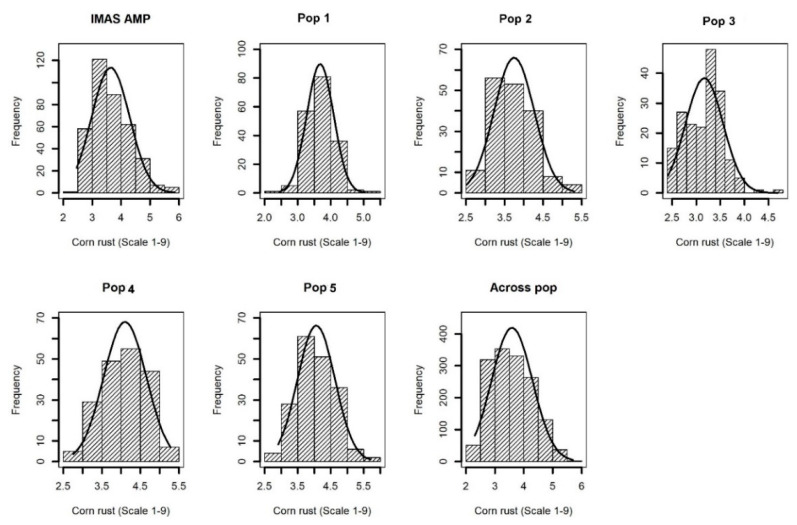
Phenotypic distribution for corn rust or common rust severity in the IMAS association panel, five F_3_ populations and across F_3_ populations.

Comparable disease pressure was observed in each environment as indicated by significant genotypic variance at each environment for each population (Table S1). Further, significant (*p* < 0.05) Pearson correlations were also observed among phenotypic values determined at different environments for each population (Table S2). This suggested that there was enough CR disease pressure in each environment. Cross-environment analyses revealed normal distributions of CR disease severity (score 1–9) in the F_3_ populations and IMAS association panel, ranging from high resistance to moderate susceptibility with scores ranging from 2.0 to 6.0 and with average means ranging from 3.4 to 4.1 for different populations (Table 1 and Figure 1). Analysis of variance for the biparental populations and the association panel showed significant genotypic and genotype x environment (GXE) variances except for GXE of Pop1 and Pop4 (Table 1). Heritability (*h^2^*) estimates were moderate with range of 0.37–0.45 for the individual F3 populations, 0.45 across the F3 populations and 0.65 for the IMAS panel.

**Figure 2:**
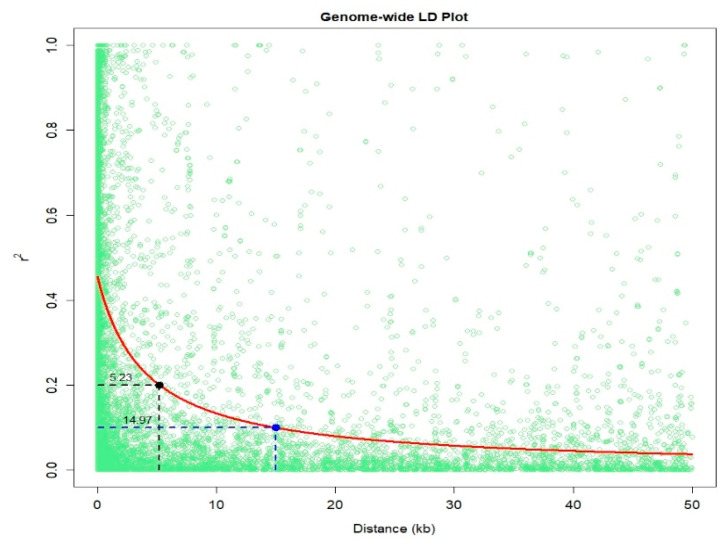
Linkage disequilibrium (LD) plot representing the average genome-wide LD decay in the panels with genome-wide markers. The values on the Y-axis represents the squared correlation coefficient r^2^ and the X-axis represents the physical distance in (kb).

**Figure 3:**
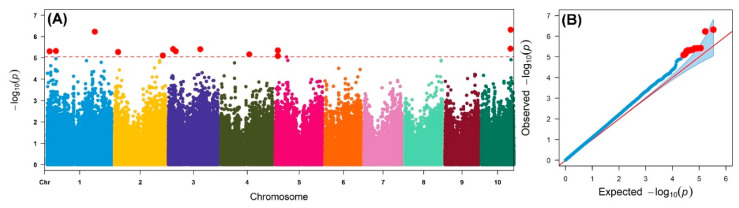
(**A**) Manhattan and q–q plots for the GWAS of common rust for the IMAS association mapping panel. The dashed horizontal line of the Manhattan plot depicts the significance threshold (*p* < 9 × 10^−6^). The *X*-axis indicates the SNP location along the 10 chromosomes, with chromosomes separated by different colors. (**B**) The red line on the q–q plot indicates a line of best fit with trait significance determined by a plot of expected-log_10_ (*p*) against observed-log_10_ (*p*).

An IMAS association mapping panel was earlier used to study the maize lethal necrosis resistance [20], where the population structure was reported in detail. In the IMAS panel, a rapid linkage disequilibrium (LD) decay across physical distance in kb was reported (Figure 2). At LD cut-off points of r^2^ = 0.1, the average physical distance was 14.97 kilo base pairs. GWAS was performed using a mixed linear model by integrating population structure (PCA) and family relatedness (kinship) within the IMAS panel using 337,110 high quality SNPs. GWAS for CR identified 14 significant marker-trait associations (MTA, significant threshold *p*  <  9 × 10^−6^). These SNPs were found across all chromosomes except on chr 6, 7, and 8 (Table 2 and Figure 3). The distribution of SNPs across chromosomes and their level of significance for CR are shown in a Manhattan plot (Figure 3A). To test the ability of the model used, a quantile-quantile plot of the observed-log *p*-value vs. the expected-log *p*-value was plotted. As shown in Figure 3B, the population structure was controlled well by the mixed linear model (Figure 3). In terms of the percentage of phenotypic variance explained (PVE), the SNPs identified from GWAS individually explained 6–10% (Table 2). SNP *S5_51353429* had the largest significant MTA while *S3_21856582* explained the most phenotypic variance. Candidate genes were selected around the significant SNPs and identify the putative function of these genes. Six candidate genes were identified in the significant SNP sites or adjacent to these sites (Table 2). We identified two candidate genes each on chromosomes 2, 5, and 10.

Linkage map for each of the five populations was constructed. For each population, the number of progenies or families, markers, map lengths, and average genetic distances between the markers are presented in Table S3. Detection of QTL in the F_3_ populations revealed seven QTLs in pop1, nine QTL in pop2, four QTL in pop3, five QTL in pop4 and one QTL in pop5, distributed on all chromosomes except chromosome 10 (Table 3). In pop1, QTLs individually explained 3–7% of phenotypic variance, in pop2, individual QTL explained 2–11% of total variation. In pop3, each QTL explained 5–9% of variation, whereas the range was 3–20% in pop4 and 17% in pop5. The total variance explained by each population ranged from 14 to 40%. Comparison of QTL detected among five populations revealed that QTL *qCR1-78* was detected in both pop1 and pop5 on chromosome 1. Another QTL *qCR3-151* detected in pop2 was within the confidence interval of the QTL *qCR3-113* detected in pop1 (Table 3). QTL *qCR9-117* detected in pop3 overlapped with QTL *qCR9-118* observed in pop2.

**Figure 4:**
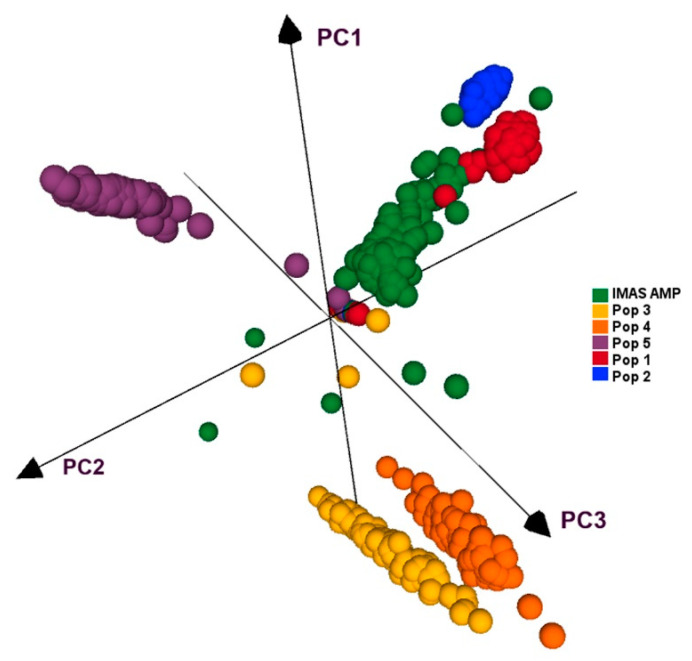
PCA analyses of five F3 populations and the IMAS panel based on GBS markers.

All five F_3_ populations and IMAS panel was plotted by using first three principal components, which together contributed for 27.5% of the variation. PCA plot showed clear clustering of the five F_3_ populations and IMAS panel into five clusters as shown in Figure 4. PCA distribution suggests pop 1 and pop 2 are more related to the IMAS panel compared to pop 3, 4, and 5. Joint linkage association mapping across five F_3_ populations constituted 921 families, identified eighteen QTLs distributed in all chromosomes except on chromosome 8 (Table 4). These QTLs individually explained 0.3 to 3.1% and together explained 45% of the total phenotypic variance. Among the 18 QTL detected through JLAM, six QTLs, *qCR1-78, qCR1-227, qCR3-172, qCR3-186, qCR4-171*, and *qCR7-137* were also detected in individual population-based linkage mapping (Table 3). Out of these 18 QTLs, one QTL on chromosome 7 (*qCR7-10*) followed by QTL on chromosome 1 (*qCR1-78*) were strongly associated with CR disease severity in terms of *p* values.

**Figure 5:**
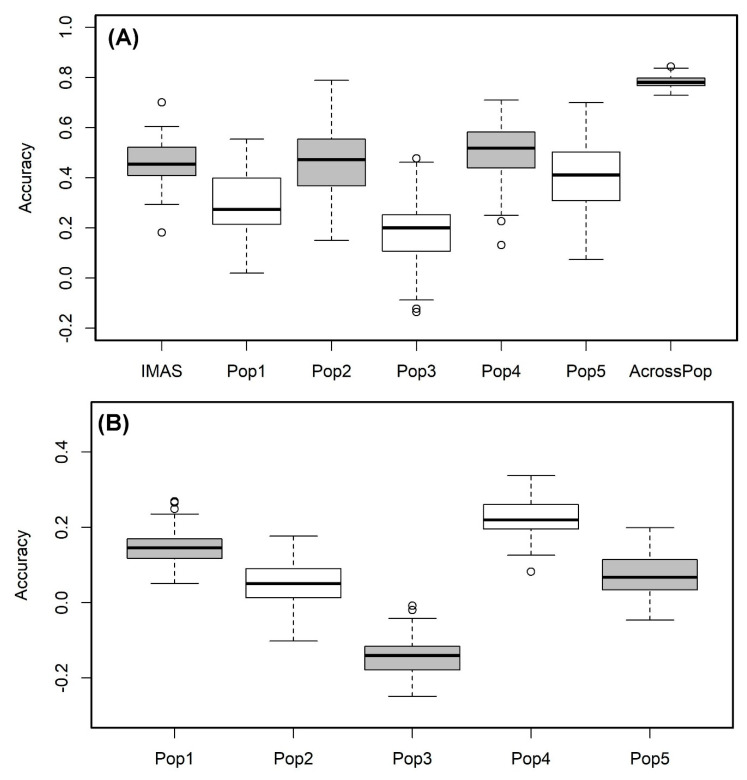
Genome-wide prediction correlations for CR resistance in bi-parental and IMAS AM panel based on two different scenarios. Scenario (**A**) estimation and prediction within IMAS AM panel and biparental populations and combined F_3_ populations; Scenario (**B**): prediction of each F_3_ population using IMAS AM panel as a training set with five-fold cross-validation.

We used the RR-BLUP model to predict the lines performance within each population. The prediction correlation was highest for pop4 (r = 0.51) followed by the IMAS panel (r = 0.46) and pop 2 (r = 0.46) and was low for pop3 with 0.19 (Figure 5A). However, the prediction through combined analysis of the five F_3_ populations reported high improvement in the prediction accuracy with 0.78 for CR (Figure 5A). Prediction of biparental populations by using IMAS panel as training population reveals the correlations of 0.15, 0.05, −0.14, 0.22 and 0.07 for pop1, pop2, pop3, pop4, and pop5, respectively (Figure 5B).

## 3. Discussion

Over the past years, CR epidemics have mainly resulted from high levels of susceptibility among commercial maize hybrids [3]. Past efforts to exploit genetic resistance for CR have largely been through R genes [9,11]. A major drawback with genetic resistance through R genes is that it is overcome with time [12]. Quantitative resistance is by far a better option compared to single gene based resistance; however, few studies have been carried out on quantitative resistance to CR. This study therefore aimed to detect quantitative resistance to CR through GWAS by using IMAS association mapping panel, and QTL detection, JLAM, and GP using five F_3_ populations.

Phenotypic analyses revealed normal distributions in all populations varied from highly resistance to moderate susceptibility, therefore indicating polygenic resistance in the materials used. Genotype and GxE interaction variances were significant in most populations which showed their importance in CR resistance. Heritability estimates were moderate in F_3_ populations. High heritability observed in the IMAS association panel may be attributed to the large genetic diversity of the germplasm used.

Previous studies on the population structure of the IMAS association panel used in the present GWAS showed confounding structure in the panel posing a need to account for it [18,35]. The quantile–quantile plot of expected vs. observed log *p* values indicated that the mixed model used in this study effectively accounted for the population structure and kinship matrix. Ideally, the *p* values are expected to follow the diagonal plot of the expected vs. observed *p* values assuming no marker trait associations. In real datasets, the markers are expected to deviate to the left side of the plot as a result of true marker trait associations, however, strong deviations to the left indicate inflated false positives while to the right indicate deflated false negatives [25].

Association mapping resolution also strongly depends on the LD of the particular population under study since it exploits the historical recombinations that occur in natural populations [36]. Rapid LD decay as shown in the IMAS population shows the significant diversity in the panel and its suitability for GWAS. The small mapping distance of 50kb and rapid decay at r^2^ = 0.1 enable the detection of marker trait associations with small effects that might have otherwise been missed with other mapping approaches. 

In the GWAS, 14 significant SNPs were associated with *P. sorghi* resistance across three environments (Table 2, Figure 3), of which some overlapped with previously reported QTL intervals [12,31,37]. Specifically, SNP *S3_21856582* detected in this study is also reported earlier [12] and interestingly it is also overlapped with two biparental based QTL studies [10,13] which suggests the possibility of potential candidate gene/marker for CR resistance across genetic backgrounds and environments. Another two SNPs on chromosome 5, *S5_10087070* and *S5_10089138* are found close to SNP S5_10,055,423 found in the previous association mapping study in tropical maize germplasm [31]. Other SNPs detected in this study seems to be novel and add for the new source of resistance for CR. 

The candidate genes in the present study were identified as encoding transcription factors (TFs), ATP binding and intracellular signaling. A putative candidate gene GRMZM2G181030 has been linked with the MYB superfamily which is one of the three largest TF families in maize [38]. The important roles of MYB superfamily are in developmental processes and defense responses in plants [39]. GRMZM2G322582 was encoded as ATP binding protein which play important roles in membrane transport, cellular motility and regulation of various metabolic processes [40]. GRMZM2G086484 was linked to the Pleckstrin homology (PH) domain superfamily protein which plays a role in recruiting proteins to different membranes, thus targeting them to appropriate cellular compartments or enabling them to interact with other components of the signal transduction pathways [41]. GRMZM2G181002 was annotated in protein kinase superfamily specifically the phosphotransferases, serine and threonine specific kinase superfamily. Serine/threonine kinase receptors play a role in the regulation of cell proliferation, programmed cell death (apoptosis), cell differentiation, and embryonic development [42]. Lastly, GRMZM2G009188 was encoded as 11-beta-hydroxysteroid dehydrogenase 1B whose role is in the regulation of energy metabolism and immune system by locally reactivating glucocorticoids predominantly by catalyzing the reduction of cortisone to cortisol in intact cells that also express hexose-6-phosphate dehydrogenase (H6PDH), which provides cofactor NADPH [43]. 

The linkage analysis within the individual bi-parental populations was also carried out revealing 23 QTLs associated with CR resistance in maize and were distributed on all chromosomes (Table 3). Linkage groups conferring CR resistance have been previously reported on bin 1.05, 1,06, 2.05, 3.04, 5.02, 6.04, 8.03, 8.05, 10.1, 10.5, and 10.6 [7,10,12,13,31]. The present study’s results corroborate these earlier studies, as most of the identified QTLs fell in those bins of linkage groups. Within the current study we also found several QTLs overlapping across populations with linkage mapping, across methods with linkage analyses, JLAM, and GWAS (Table 2, Table 3, Table 4). QTL *qCR1-139* detected on pop4 was co-located with QTL detected in F_3_ pop 3 and F_3_ pop 5, which suggests one of possible potential candidate for CR resistance in chromosome 1 across different genetic back grounds.

Identification of genomic regions for CR resistance have previously been carried out in biparental-based linkage analysis [7,10,13,30] and natural population-based association mapping [12,31]. The current study consequently sought to exploit JLAM using multiple segregating populations in conjunction with QTL analysis and GWAS. GWAS and JLAM increased the resolution within the confidence intervals of QTL for CR resistance. *qCR1-139*, which explained >20% of the phenotypic variance, was a locus with major effect based on genetic linkage mapping, and was likely to consist of two QTL as revealed by GWAS and JLAM results. SNP *S1_220067760* identified through GWAS and SNP *S1_227241027* identified through JLAM fall within the confidence interval of QTL *qCR1-139* (Table 2, Table 3 and Table 4). The significant marker *S2_16361185* detected through GWAS and marker *S2_20589802* (*qCR2-20*) detected through JLAM fall within the confidence interval of another QTL on chromosome 2, *qCR2-16* detected in pop3, which helps to increase the precision of QTL position. Fractionation of single major QTL was also reported previously in maize [44]. Thus, the combination of linkage mapping, JLAM and GWAS approaches helped to further refine the CR resistance loci to an extent that it became possible to separate the effects of two co-segregating QTL (on chromosome 1 and 2). Thus, GWAS and JLAM can serve as a great complementary tool to identify and validate the molecular markers linked to QTL and to be used in applied breeding. Nevertheless, it is warranted to validate through fine mapping and gene cloning coupled with functional genomics studies, in order to clarify further on QTL intervals identified and refined in this study.

Since GS was demonstrated to be useful in plant breeding, there have been many studies that demonstrate the utility of GP in breeding for disease resistance in crops [26,45,46,47,48,49]. Prediction correlations for within association panel and biparental populations is comparable to the other disease reported in maize [18,24,50]. As described in earlier study [51], the benefits of large populations or combined population predictions were higher compared to individual population-based predictions. This corroborates with the present study whereby combined population prediction was higher over individual or smaller population-based predictions (Figure 5A). 

The success of GP gains more attention when the training population is related but independent of testing or validating populations. In the current study, since the parental lines of F_3_ populations are part of the IMAS association panel, we tried to use IMAS panel as an independent training population and predict the F_3_ populations for their performance for CR resistance. We observed, for four out of five populations, prediction correlations of 0.05, 0.07, 0.15, and 0.22 (Figure 4), which is comparable to the prediction correlations observed for maize lethal necrosis [26] under similar testing environments. Overall, results from our study encourages to develop an independent but related diverse population as training population to predict CR resistance. This also supports the use of GEBVs as indirect selection criteria, at least to remove the lines with poor performance for CR resistance. Conducting separate field trials for CR screening in the breeding pipelines always takes additional resources. The model’s predictive ability, with cross validation, showed moderate prediction accuracies for CR in pop1 and pop4 were comparable to the correlations observed under similar scenario of predicting biparental populations by using association mapping panel for maize lethal necrosis and maize chlorotic mottle virus resistance [26]. Overall, to have an effective independent training population, relatedness between training and validation population is critical as well as the trait architecture and heritability. Compared to abiotic stress related traits, traits like CR and many other maize diseases are relatively less complex and our results suggests that it is possible to have an independent training population with continuous improvement and used routinely in breeding program.

In conclusion, we used five F3 populations and one IMAS association mapping panel, together comprising 1300 lines, to unravel the genetic architecture of CR resistance. In this study we identified new QTLs as well as reconfirmed the QTLs reported in earlier studies for CR resistance in tropical and subtropical maize germplasm. Linkage mapping identified 26 QTLs with four major QTLs, which explained >10% of phenotypic variance. The detected QTLs were validated with GWAS, and several SNPs were found overlapping with the identified QTLs through either linkage mapping or JLAM. These genomic regions can serve as potential sources to improve resistance to CR. GP can be used in combined populations to predict the response of the germplasm to CR resistance. Having a common training population derived from intensively phenotyped and genotyped lines with diverse representation from a breeding program holds promise in breeding for CR resistance.

## 4. Materials and Methods 

### 4.1. Plant Materials and Trial Design

A collection of 380 tropical and subtropical maize inbred lines denoted as the Improved Maize for African Soils (IMAS) panel, representing some of the genetic diversity available in CIMMYT’s and several national research programs from Africa and Mexico breeding programs (low N, drought, and biotic stresses) was used in this study [26,35]. In addition five F_3_ populations namely Pop1 (CZL0618 × LaPostaSeqC7-F71-1-2-1-1B, *n* = 183), Pop2 (CZL074 × LaPostaSeqC7-F103-1-2-1-1B, *n* = 174), Pop3 (CZL00009 × CZL99017, *n* = 187), Pop4 (CML505 × CZL99017, *n* = 189) and Pop5 (CZL0723 × CZL0724, *n* = 188) were used in this study [52]. These five F_3_ populations were used to study the grain yield under optimum and drought stress conditions [52,53]. The IMAS panel was evaluated in three location-year combinations, at Kitale (1.0191° N 35.0023° E, 1900 meters above sea level (masl)) in 2013 and 2014 and at Kakamega (0°17’3.19” N 34°45’8.24” E, 1535 masl) in 2014, and F_3_ pop 2 was evaluated in three locations Kakamega, Kitale, and Embu (0°31′52″ S 37°27′02″ E, 1406 masl) and the remaining four F_3_ populations were evaluated in two locations in Kakamega and Embu at 2011 in Kenya (Tables S1 and S2).

**Figure 6:**
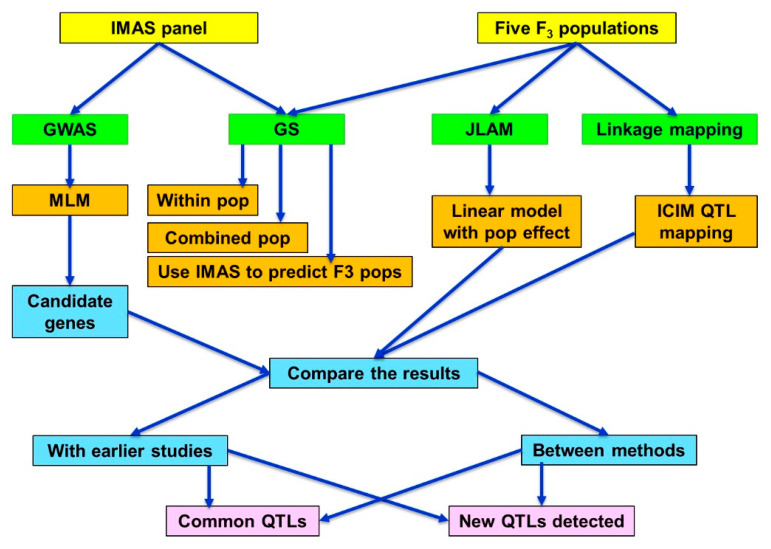
Outline of the general flow chart on population used in this study and the analyses methods applied. GWAS—Genome-wide association study, GS—Genomic selection, JLAM—Joint linkage association mapping, MLM—mixed linear model, ICIM—Inclusive composite interval mapping.

All the lines from IMAS panel and five F_3_ populations were planted in a 4-m-long single row plots in an alpha lattice design with two replications in each location. Two seeds were planted per hill and thinned to a single plant per hill, three weeks after emergence to ensure uniform plant density. Standard agronomic practices were followed. The chosen locations were natural hotspots for foliar diseases including CR; good disease infection pressure across the trials at each location was observed. The IMAS panel, and F_3_ populations were evaluated for their responses to CR in two to three environments (Tables S1 and S2). CR disease severity data was recorded after flowering and scored plot-wise on an ordinal scale of 1 (no rust, highly resistant, without disease symptoms) to 9 (highly susceptible, most severe). On the IMAS panel, in addition to CR severity scoring, data were also collected for anthesis date (AD). The populations used in this study, methods applied, and the different type of analyses used in this study are presented as a general analyses diagram (Figure 6).

### 4.2. Phenotypic and Genotypic Data Analysis

For IMAS panel and each F_3_ populations, each location-year combinations were treated as an independent environment which resulted into three environments for IMAS panel, and F_3_ pop2, whereas the number of environments were two for F_3_ pop1, F_3_ pop3, F_3_ pop4, and F_3_ pop5. Analysis of variance for individual and across environments was undertaken using the ASREML-R [54] for each bi-parental population and the IMAS panel. The following statistical model was used to estimate the variance components (Equation (1):

*CR_mnop_* = µ + Genotype*_m_* + Env*_n_* + (Genotype x Env)*_mn_* + Rep(Env)*_on_* + Block(Rep.Env)*_pno_* + e*_mnop_* (1)

where CR*_mnop_* is the phenotypic performance of the *m*th genotype at the *n*th environment in the *o*th replication of the *p*th incomplete block, *µ* is an intercept term, *Genotype_m_* is the genetic effect of the *m*th genotype, *Env_n_* is the effect of the *n*th environment, (*Genotype x Env*)*_mn_* is the interaction effect between genotype and environment, *Rep(Env*)*_on_* is the effect of the *o*th replication at the *n*th environment, *Block(Rep.Env*)*_pno_* is the effect of the *p*th incomplete block in the *o*th replication at the *n*th environment, and e*_mnop_* is the residual. The genotypic effect, genotype by environment interaction and effect of incomplete blocks were treated as random effects in order to estimate their variances and residual error variance. Environments and replications were treated as fixed effects. Assuming fixed genotypic effects, a mixed linear model was fitted to obtain the best linear unbiased estimates (BLUEs). Significance of variance components were tested by model comparison with likelihood ratio tests in which the halved *p*-values were used as an approximation. Broad-sense heritability (H^2^) was calculated for all the traits using the following Equation (2):

(2) $$H^{2}\;=\;\frac{\sigma_{g}^{2}}{\sigma_{g}^{2}+\;\frac{\sigma_{\mathrm{gl}}^{2}}{l}+\;\frac{\sigma_{\varepsilon }^{2}}{\mathrm{lr}}}$$

where σ^2^*_g_* is the genotype variance; σ^2^*_gl_* is the genotype × environment interaction variance; and σ^2^_Ɛ_ is the error variance, *l* represents number of environments and *r* for number of replications. META-R software [55] was used to obtain best linear unbiased prediction (BLUP) for each genotype across environments. Combined analyses of the five F_3_ populations were carried out in META-R. 

The F_3_ populations lines as well as their parents, and the IMAS association panel inbred lines were genotyped with Genotyping by Sequencing (GBS). DNA of all lines was extracted from 3–4 leaves stage seedlings and genotyped using GBS platform at the Institute for Genomic Diversity, Cornell University, Ithaca, USA as per the procedure described in earlier studies [56]. The~955K GBS SNP datasets were filtered where a minor allele frequency of <0.05, heterozygosity of >5% and missing data rates >10% were excluded from further analysis in TASSEL ver 5.2 [57].

### 4.3. PCA and Linkage Disequilibrium

The principal components (PC) of the five F_3_ populations and IMAS panel were estimated using TASSEL ver 5.2 and visualized using R software version 3.2.5 (https://www.r-project.org/) to obtain the explained variance of each PCs. Further, the LD of the IMAS panel was calculated using Tassel version 5.2 to obtain the rate of LD decay in the population. LD decay rate between each pair of SNPs was analyzed with the squared Pearson correlation coefficient (r^2^). The rate of LD decay with physical distance was visualized and average pairwise distances at which LD decayed at r^2^ = 0.1 and 0.2 were calculated in R software. The ‘nlin’ function in R was used to fit non-linear models into the genome-wide LD data by incorporating r^2^ as responses (y axis) and pairwise distances (x axis) as predictors. The average estimator for LD decay was calculated at ‘significance’ threshold of r^2^ = 0.1 and r^2^ = 0.2 cutoff points in relation to distance [58] and a representative scatter plot was drawn as LD between adjacent markers versus chromosome distance (kb).

### 4.4. Genome-wide Association Analyses

The BLUP values obtained for CR were used in GWAS as phenotypes. The kinship matrix obtained with a centered identity by state (IBS) and the first five PCs which explained maximum variation were used to correct the population structure in a mixed linear model using TASSEL version 5.2. Genome wide scans for marker-trait associations were conducted with mixed linear model. The amount of phenotypic variation explained by the model was assessed using the R^2^ statistics, calculated by fitting all significant SNPs simultaneously in a linear model in R. To determine the significance threshold, multiple testing correction was conducted where the total number of tests were estimated based on the average extent of LD at r^2^ = 0.1 [19]. With respect to the above, significant associations were declared when *p* values in independent tests were less than 9 × 10^−6^. The 50 bp source sequences of the significantly associated SNPs were used to perform BLAST searches against the B73 RefGen v2 genome set in Maize GDB (http://www.maizegdb.org). The putative candidate genes identified in Maize GDB were within or adjacent to each associated SNP.

### 4.5. Detection of QTLs and Joint Linkage Association Mapping

The number of SNPs was further reduced by selecting homozygous and polymorphic markers between the parents in each population and were further filtered based on minimum distance between adjacent SNPs as ≥ 200 kilo base pairs (Kbps). For JLAM, markers from all five F_3_ populations were combined, and markers with <1% missing value and >5% MAF and heterozygosity of <5% were retained. Finally, a set of 5000 SNPs that are uniformly distributed across the genome were used for JLAM analyses.

QTL IciMapping version 4.1 [59] was used to construct the linkage map based on data from all five biparental populations. QTL IciMapping was used to remove the highly correlated SNPs that do not provide any additional information by using an inbuilt tool BIN. This resulted in retention of 1130, 1047, 1099, 1122, and 1081 high-quality SNPs in pop1, pop2, pop3, pop4, and pop5, respectively (Table S3). BLUP values across environments for the F_3_ populations were used in QTL detection analysis using inclusive composite interval mapping (ICIM). The probability in the stepwise regression was set at 0.01 and the scanning step was 1 cM. For determination of QTL significance, the threshold LOD score was set to >2.5 by using 1000 permutations and a *p* value ≤ 0.05. The phenotypic variation explained (PVE) by each QTL and across all QTLs for each trait was estimated. The origin of the favorable allele for CR resistance was identified based on the sign of the additive effects of each QTL. 

A set of 5000 high-quality GBS SNPs derived from the genotyped F_3_ populations were used in JLAM analyses. BLUPs calculated across populations and environments were used as phenotypes. To best carry out association mapping in multiple biparental populations, a two-step biometric model which incorporates population effect, cofactors and a marker effect across populations was used to detect QTL [60,61]. This model was explained in detail by Liu et al. [61] and Würschum et al. [60]. The first step involved selection of cofactors based on Schwarz Bayesian Criterion (SBC) [62] by including a population effect and cofactors carried out using PROC GLM SELECT implemented in the statistical software SAS 9.4 [63]. In the second step, *p* values for the *F*-test were calculated using a full model (including SNP effect) versus a reduced model (without SNP effect). Genome-wide scans for QTLs were implemented in R version 3.2.5 (R Development Core Team, Vienna, Austria; 2015). The Bonferroni–Holm procedure [64] was used to detect markers with significant (*p* < 0.05) main effects and was controlled for multiple testing. The total proportion of PVE by the detected QTLs was calculated by fitting all significant SNPs simultaneously in a linear model to obtain an adjusted *R*2 [65]. 

### 4.6. Genomic Prediction

GP was carried out with ridge regression BLUP [66] within and across the five F_3_ populations as well as in the IMAS panel for CR resistance at five-fold cross-validation. BLUEs across environments for each of the biparental populations and across five F3 populations were used for the analysis. For all biparental populations and IMAS panel, same set of high-quality uniformly distributed 5000 SNPs with no missing values and MAF > 0.05 were used. Prediction scenarios used for GP include: ‘within population’ where training and validation sets are derived from within individual biparental population and IMAS panel; a ‘combined population’ prediction approach where data from five F_3_ populations were combined and sampled randomly to form validation and training set; and ‘across population’ prediction in which IMAS association panel was used as a training set and each F_3_ population as a validation population. For each approach, 100 iterations were done for sampling of the training and validation sets. 

## Figures and Tables

**Table 1:** Means, ranges, and components of variance for Common rust in an IMAS association panel, F3 populations and across F3 populations.

Population	Mean (Range)	σ^2^_G_	σ^2^_GxE_	σ^2^_e_	*h^2^*
IMAS AMP	3.80 (2.40–5.90)	0.07 *	0.03 *	0.13	0.68
CZL0618 × LaPostaSeqC7-F71-1-2-1-1B–Pop1	3.61 (2.00–5.50)	0.024 *	0.01	0.10	0.44
CZL074 × LaPostaSeqC7-F103-1-2-1-1B–Pop2	3.70 (2.50–5.30)	0.02 *	0.02 *	0.12	0.43
CZL00009 × CZL99017–Pop3	3.42 (2.32–5.00)	0.03 *	0.02 *	0.11	0.45
CML505 × CZL99017–Pop4	4.10 (2.76–5.14)	0.01 *	0.01	0.12	0.37
CZL0723 × CZL0724–Pop5	4.02 (2.51–6.02)	0.03 *	0.02 *	0.16	0.38
Across five populations	3.85 (2.02–6.00)	0.04 *	0.10 *	0.15	0.45

* Significance at *p* < 0.05.

**Table 2:** Chromosomal position and SNPs significantly associated with Common rust disease severity (DS) detected by SNP-based GWAS in the IMAS association mapping panel.

SNP ^a^	Chr	MLM-*P* Value	R^2^ (%)	MAF	Allele	Putative Candidate Genes	Predicted Function of Candidate Gene
S1_12663024	1	4.86 × 10^−6^	7.00	0.14	A/G	*GRMZM2G480386*	uncharacterized
S1_41433126	1	4.69 × 10^−6^	7.88	0.05	G/A	*GRMZM5G886521*	uncharacterized
S1_220067760	1	5.86 × 10^−7^	7.80	0.46	C/T	*GRMZM2G564469*	uncharacterized
S2_16361185	2	5.23 × 10^−6^	7.70	0.08	C/T	*GRMZM2G086484*	Pleckstrin homology (PH) domain superfamily protein
S2_222274747	2	7.65 × 10^−6^	7.10	0.05	C/T	*GRMZM2G009188*	11-beta-hydroxysteroid dehydrogenase 1B
S3_21856582	3	3.84 × 10^−6^	9.54	0.42	A/C	*GRMZM2G395983*	uncharacterized
S3_34683394	3	4.83 × 10^−6^	9.02	0.35	A/G	*GRMZM5G881063*	uncharacterized
S3_147013779	3	3.89 × 10^−6^	7.70	0.17	G/C	*GRMZM2G060540*	uncharacterized
S4_130478096	4	6.72 × 10^−6^	6.93	0.12	A/T	*GRMZM5G833902*	uncharacterized
S5_10087070	5	8.02 × 10^−6^	6.25	0.06	A/G	*GRMZM2G181002*	Phosphotransferases, Serine or threonine-specific kinase subfamily
S5_10089138	5	4.39 × 10^−6^	6.56	0.07	T/C	*GRMZM2G181002*	
S5_51353429	5	9.05 × 10^−6^	7.40	0.19	G/A	*GRMZM2G457211*	uncharacterized
S10_134585613	10	3.68 × 10^−6^	6.65	0.12	C/T	*GRMZM2G322582*	ATP binding protein
S10_134831452	10	4.78 × 10^−7^	8.22	0.14	A/G	*GRMZM2G181030*	MYB-related transcription factor family that regulates hypocotyl growth by regulating free auxin levels in a time-of-day specific manner (RVE1)

MAF—Minor allele frequency; the underlined SNP allele is minor allele; R^2^—proportion of phenotypic variance explained by SNP; ^a^ The exact physical position of the SNP can be inferred from marker’s name, for example, S5_51353429: chromosome 5; 51,353,429 bp (Ref. Gen_v2 of B73).

**Table 3:** Detection of QTL associated with resistance to Common rust, their physical positions and genetic effects in five F3 populations.

QTL Name	Chr	Position (cM)	LOD	PVE (%)	Add	Dom	Total PVE (%)	Left Marker	Right Marker
CZL0618 × LaPostaSeqC7-F71-1-2-1-1B–Pop1									
*qCR1-78*	1	551	4.41	3.32	−0.20	−0.08	14.95	**S1_77801418**	**S1_80167797**
*qCR1-290*	1	799	2.82	3.59	0.18	−0.13		S1_290957469	S1_285979058
*qCR2-198*	2	187	3.17	6.70	−0.34	−0.53		S2_198394488	S2_230388748
*qCR3-113*	3	307	2.85	6.50	0.26	−0.24		**S3_224567900**	**S3_113425715**
*qCR6-38*	6	194	2.79	3.62	0.21	−0.01		S6_37902339	S6_63537451
*qCR6-63*	6	197	4.51	3.27	−0.21	−0.02		S6_63537451	S6_65299800
*qCR6-146*	6	446	3.09	7.03	0.30	−0.42		S6_147225115	S6_146382028
CZL074 × LaPostaSeqC7-F103-1-2-1-1B–Pop2									
*qCR2-137*	2	414	2.54	2.38	0.00	0.18	39.5	S2_158674609	S2_136562142
*qCR3-8*	3	262	3.33	2.97	0.63	0.52		S3_8300745	S3_8888914
*qCR3-151*	3	405	6.56	6.28	0.17	−0.14		**S3_150831482**	**S3_166811360**
*qCR4-198*	4	351	2.58	5.64	−0.19	−0.05		S4_197820294	S4_200964285
*qCR4-198*	4	354	5.77	8.85	0.24	#x2212;0.01		S4_200964285	S4_198430250
*qCR5-51*	5	374	2.93	3.7	−0.17	0.27		S5_186678634	S5_51355494
*qCR8-123*	8	165	4.69	6.23	0.20	0.00		S8_130213071	S8_123469991
*qCR9-118*	9	291	2.55	8.46	−0.23	0.03		**S9_120748383**	**S9_118065757**
*qCR9-12*	9	359	5.63	10.95	−0.28	−0.03		S9_12599819	S9_11929364
CZL00009 × CZL99017–Pop3									
*qCR1-18*	1	394	3	5.21	−0.13	−0.08	12.99	S1_19328973	S1_17679542
*qCR1-172*	1	501	2.81	6.09	0.67	−0.6		**S1_196052894**	**S1_171534815**
*qCR2-16*	2	93	3.61	5.84	0.7	−0.47		S2_16401968	S2_181538947
*qCR9-117*	9	300	4.31	8.35	−0.18	−0.04		**S9_122035011**	**S9_116948078**
CML505 × CZL99017–Pop4									
*qCR1-139*	1	182	3.87	20.16	0.32	0.67	23.89	**S1_139463362**	**S1_227241027**
*qCR4-171*	4	239	4.83	5.55	−0.23	0.01		S4_171215058	S4_173802342
*qCR7-137*	7	87	9.79	11.01	−0.34	0.22		S7_140894965	S7_137169719
*qCR9-47*	9	410	3.03	2.79	0.11	−0.24		S9_47064183	S9_58143264
*qCR9-90*	9	432	3.18	3	−0.13	−0.2		S9_90366846	S9_97737243
CZL0723 × CZL0724–Pop5									
*qCR1-77*	1	89	6.52	17.39	0.29	−0.09	14.28	**S1_73375502**	**S1_77145631**

Chr—Chromosome; LOD—logarithm of odds; Add—additive effect; Dom—dominance effect; PVE—phenotypic variance explained; Markers with bold letters are the QTL consistent in at least two across populations.

**Table 4:** Analysis of trait-associated markers, allele substitution (α) effects, and the total phenotypic variance (R^2^) of the joint linkage association mapping based on combined 8 F3 populations.

Marker	QTL_Name ^a^	Chrom	Pos	α-effect	*p* Value	PVE (%)	*P_G_*
**S1_77801418**	*qCR1_78*	1	77.80	0.16	1.34 × 10^−12^	2.9	7.2
**S1_227241027**	*qCR1_227*	1	227.24	0.17	3.75 × 10^−7^	1.5	3.8
S2_20589802	*qCR2_20*	2	205.90	0.11	1.51 × 10^−3^	0.6	1.5
**S3_172332492**	*qCR3_172*	3	172.33	−0.06	1.50 × 10^−2^	0.3	0.7
**S3_186725598**	*qCR3_186*	3	186.73	0.10	9.88 × 10^−3^	0.4	1
S4_828312	*qCR4_1*	4	0.83	−0.19	6.02 × 10^−10^	2.2	5.5
S4_5238963	*qCR4_5*	4	5.24	0.12	7.45 × 10^−8^	1.6	4
**S4_171215058**	*qCR4_171*	4	171.22	−0.05	3.79 × 10^−2^	0.2	0.5
S5_2363546	*qCR5_2*	5	2.36	−0.06	2.03 × 10^−2^	0.3	0.7
S6_32969273	*qCR6_32*	6	32.97	0.10	4.49 × 10^−5^	0.9	2.2
S6_144280146	*qCR6_144*	6	144.28	0.06	2.57 × 10^−2^	0.3	0.7
S6_154981658	*qCR6_155*	6	154.98	−0.19	8.37 × 10^−4^	0.6	1.5
S7_10651847	*qCR7_10*	7	10.65	−0.28	1.78 × 10^−13^	3.1	7.8
S7_13389227	*qCR7_13*	7	133.89	0.20	3.25 × 10^−11^	2.5	6.2
**S7_137335046**	*qCR7_137*	7	137.34	−0.14	3.26 × 10^−9^	2	5
S9_134919722	*qCR9_135*	9	134.92	−0.14	5.08 × 10^−5^	0.9	2.2
S10_132612571	*qCR10_132*	10	132.61	−0.15	2.76 × 10^−9^	2	5
S10_133744261	*qCR10_133*	10	133.74	−0.09	1.79 × 10^−3^	0.5	1.2

^a^ QTL name composed by the trait code followed by the chromosome number in which the QTL was mapped and a physical position of the QTL, Chr—Chromosome, PVE—phenotypic variance explained, *P_G_*—genotypic variance explained, The marker name indicates chromosome number followed by its physical position, for example S6_68207704 represents marker is in chromosome 6 at a position of 68,207,704bp (Ref. Gen_v2 of B73). Markers with bold letters are the QTL consistent with JLAM and linkage mapping.

## Supplementary Materials

The following are available online at https://www.mdpi.com/1422-0067/21/18/6518/s1.
