# Supplementary material for: Combination of Linkage Mapping, GWAS, and GP to Dissect the Genetic Basis of Common Rust Resistance in Tropical Maize Germplasm
Source: Int J Mol Sci. 2020 Sep 6;21(18):6518. doi: 10.3390/ijms21186518 (PMC7555316; doi:10.3390/ijms21186518)
Supplement: Supplementary file 1 [file ijms-21-06518-s001.pdf]

Supplementary material

Supplementary Table S1: Mean and variance components for GLS disease severity in each location for IMAS AM panel and five biparental populations

IMAS AM panel	Mean	σ^2_G	σ^2_e	h^2	LSD	CV
Kitale2013	3.05	0.15*	0.16	0.66	0.47	26.95
Kitale2014	3.80	0.14*	0.17	0.61	0.46	24.03
Kakamega2014	3.80	0.02*	0.04	0.41	0.19	9.60
CZL0618 x LaPostaSeqC7-F71-1-2-1-1B - Pop1						
Kakamega2011	4.21	0.04*	0.14	0.35	0.31	14.41
Embu 2011	2.70	0.02*	0.05	0.50	0.11	21.45
CZL074xLaPostaSeqC7-F103-1-2-1-1B F3pop2						
Kakamega2011	3.70	0.04*	0.19	0.29	0.33	19.04
Kitale2011	2.50	0.02	0.11	0.27	0.16	27.01
Embu 2011	4.12	0.07*	0.15	0.49	0.38	15.48
CZL00009 x CZL99017 – Pop3						
Kakamega2011	4.30	0.03*	0.19	0.25	0.31	20.90
Embu 2011	2.47	0.04*	0.17	0.35	0.11	12.96
CML505 x CZL99017 – Pop4						
Kakamega2011	3.12	0.02*	0.11	0.27	0.09	21.66
Embu 2011	2.47	0.11*	0.25	0.46	0.48	17.21
CZL0723 x CZL0724 – Pop5						
Kakamega2011	4.27	0.05*	0.19	0.35	0.36	17.64
Embu 2011	3.22	0.03*	0.16	0.27	0.19	29.16

* P = 0.05

Supplementary Table S2: Phenotypic correlations among locations for common rust resistance in IMAS AM panel and five F3 populations

IMAS AM panel	Kitale2013	Kitale2014
Kitale2013	0.90*	
Kakamega2014	0.60*	0.71*
CZL074xLaPostaSeqC7-F103-1-2-1-1B F3pop2	Kitale2011	Embu2011
Kakamega2011	0.56*	0.51*
Embu2011	0.63*	NA
CZL0618 x LaPostaSeqC7-F71-1-2-1-1B - Pop1	Embu2011	
Kakamega2011	0.59*	
CZL00009 x CZL99017 – Pop3	Embu2011	
Kakamega2011	0.68*	
CML505 x CZL99017 – Pop4	Embu2011	
Kakamega2011	0.52*	
CZL0723 x CZL0724 – Pop5	Embu2011	
Kakamega2011	0.46*	

* P = 0.05

Supplementary Table S3. Summary of the linkage groups constructed based on data from five F3 populations.

Population	No. of progenies	No. of SNPs	Map length	Avg distance (cM)
CZL0618 x LaPostaSeqC7-F71-1-2-1-1B - Pop1	183	1130	4605.2	4.07
CZL074 x LaPostaSeqC7-F103-1-2-1-1B – Pop2	174	1047	4390.8	4.19
CZL00009 x CZL99017 – Pop3	187	1099	5049.7	4.59
CML505 x CZL99017 – Pop4	189	1122	5632.3	5.02
CZL0723 x CZL0724 – Pop5	188	1081	4698.8	4.35
